# Tobacco Exposure Enhances Human Papillomavirus 16 Oncogene Expression via EGFR/PI3K/Akt/c-Jun Signaling Pathway in Cervical Cancer Cells

**DOI:** 10.3389/fmicb.2018.03022

**Published:** 2018-12-17

**Authors:** Juan P. Muñoz, Diego Carrillo-Beltrán, Víctor Aedo-Aguilera, Gloria M. Calaf, Oscar León, Edio Maldonado, Julio C. Tapia, Enrique Boccardo, Michelle A. Ozbun, Francisco Aguayo

**Affiliations:** ^1^Departamento de Oncología Básico Clínica, Facultad de Medicina, Universidad de Chile, Santiago, Chile; ^2^Center for Advanced Research, Tarapaca University, Arica, Chile; ^3^Center for Radiological Research, Columbia University Medical Center, New York, NY, United States; ^4^Virology Program, Instituto de Ciencias Biomédicas, Faculty of Medicine, University of Chile, Santiago, Chile; ^5^Programa Biología Celular y Molecular, Facultad de Medicina, Instituto de Ciencias Biomédicas, Universidad de Chile, Santiago, Chile; ^6^Department of Microbiology, Institute of Biomedical Sciences, University of Sao Paulo, São Paulo, Brazil; ^7^Department of Molecular Genetics and Microbiology, The University of New Mexico School of Medicine, Albuquerque, NM, United States; ^8^Advanced Center for Chronic Diseases (ACCDiS), Faculty of Medicine, University of Chile, Santiago, Chile

**Keywords:** cervical cancer, papillomavirus, cigarette smoke, signaling, HPV oncoproteins

## Abstract

High-risk human papillomavirus (HR-HPV) infection is not a sufficient condition for cervical cancer development because most infections are benign and naturally cleared. Epidemiological studies revealed that tobacco smoking is a cofactor with HR-HPV for cervical cancer initiation and progression, even though the mechanism by which tobacco smoke cooperates with HR-HPV in this malignancy is poorly understood. As HR-HPV E6/E7 oncoproteins overexpressed in cervical carcinomas colocalize with cigarette smoke components (CSC), in this study we addressed the signaling pathways involved in a potential interaction between both carcinogenic agents. Cervical cancer-derived cell lines, CaSki (HPV16; 500 copies per cell) and SiHa (HPV16; 2 copies per cell), were acutely exposed to CSC at various non-toxic concentrations and we found that E6 and E7 levels were significantly increased in a dose-dependent manner. Using a reporter construct containing the luciferase gene under the control of the full HPV16 long control region (LCR), we also found that p97 promoter activity is dependent on CSC. Non-synonymous mutations in the LCR-resident TPA (12-O-tetradecanoylphorbol 13-acetate)-response elements (TRE) had significantly decreased p97 promoter activation. Phosphoproteomic arrays and specific inhibitors revealed that CSC-mediated E6/E7 overexpression is at least in part reliant on EGFR phosphorylation. In addition, we showed that the PI3K/Akt pathway is crucial for CSC-induced E6/E7 overexpression. Finally, we demonstrated that HPV16 E6/E7 overexpression is mediated by JUN. overexpression, c-Jun phosphorylation and recruitment of this transcription factor to TRE sites in the HPV16 LCR. We conclude that acute exposure to tobacco smoke activates the transcription of HPV16 E6 and E7 oncogenes through p97 promoter activation, which involves the EGFR/PI3K/Akt/C-Jun signaling pathway activation in cervical cancer cells.

## Introduction

Human papillomaviruses (HPV) are small and naked DNA viruses with tropism for squamous stratified epithelia where it replicates and establishes either acute and persistent infections (zur Hausen, 2002). Around 12 HPV types, so-called high-risk HPV (HR-HPV), are etiologically related to cervical, anogenital, oropharyngeal and oral cancers (Serrano et al., 2017). Cervical cancer is the fourth most prevalent cancer among women worldwide (Schiffman et al., 2007; Jemal et al., 2013). Almost 100% of cervical carcinomas are etiologically related to persistent HR-HPV infection and approximately 50% of them are related to the HR-HPV16 genotype (Serrano et al., 2017).

Human papillomaviruses gene expression is regulated by a long control region (LCR) which contains an enhancer motif where cellular and viral proteins bind to cognate binding sites and regulate the activity of the early promoter, located next to the E6 start codon (nucleotide 97 in HPV16 and 105 in HPV18) (Chow et al., 2010). A consensus TATA box located upstream of the transcription initiation site recruits the TFIID transcription factor. Upstream of the initiation start site, E2 viral protein binds to its cognate sites located in the early promoter (known as E2 binding sites or E2BS), repressing its activity. In addition, the transcription factors activator protein 1 (AP-1) and specificity protein 1 (SP-1) are the most important cellular regulators of HPV gene expression. Mutations in AP-1 binding sites (classically defined as the 12-O-tetradecanoylphorbol-13-acetate-responsive element or TRE sites) completely abolish HPV early promoter activity in cell lines, demonstrating the importance of AP-1 for HPV replication and gene expression (Butz and Hoppe-Seyler, 1993). The HPV early promoter is active in diverse epithelial cell lines, as previously described (Thierry, 2009). The HPV early and late proteins are translated from polycistronic and polyadenylated transcripts processed by alternative splicing (Wu et al., 2017). Specifically, The E6 and E7 early proteins are overexpressed in HR-HPV associated tumors, often attributed to the loss of the viral E2 protein, a repressor of the early p97 promoter. In addition, these proteins are able to promote cell immortalization and are strongly related to malignant transformation (Moody and Laimins, 2010). Although E6 and E7 oncoproteins, through their abilities to interact with different cellular protein partners, are required for cervical cancer development, other cofactors are necessary for establishment and progression to cancer (White et al., 2012). Both host and environmental factors are relevant for increasing the transformation properties of HR-HPV oncoproteins (Castellsagué and Muñoz, 2003). Epidemiological studies, reported that tobacco smoking women are more susceptible to cervical cancer in the presence of HR-HPV infection (Giuliano et al., 2002; Matsumoto et al., 2010; Fonseca-Moutinho, 2011). Besides, it was reported that tobacco smoke decreases the titers of HPV antibodies, favoring future HPV infections (Kelsey et al., 2015). Thus, tobacco smoking is a recognized cofactor for cervical carcinogenesis (Castle et al., 2002). However, the mechanisms by which tobacco smoking contributes to cervical carcinogenesis in the context of HR-HPV infection remain unclear. Previously, Melikian et al. (1999) described the presence of benzo[a]pyrene metabolites in cervical mucous and Alam et al. (2008) reported that incubation of cervical organotypic epithelial rafts with benzo[a]pyrene leads to an increase of HPV titer. Moreover, the same authors described that MAPK/ERK signaling is important for tobacco-smoke mediated-viral load amplification (Bowser et al., 2011). On the other hand, Wei et al. (2014), using *in vitro* models, concluded that tobacco smoke-mediated E6 and E7 overexpression occurs predominantly at early stages of cervical cancer progression. Nevertheless, signaling pathways and mechanisms involved in tobacco-smoke mediated E6/E7 up-regulation remain unclear. Here, we report that tobacco smoke induces p97 promoter activation in CaSki (HPV16, 500 copies/cell) and SiHa (HPV16, 2 copies/cell) cervical cancer-derived cells and this activation involves EGFR activation and c-Jun phosphorylation which in turn, is recruited to TRE sites on the HPV16 LCR. In addition, we found that PI3K/Akt signaling pathway is critical for tobacco smoke-mediated E6 and E7 overexpression.

## Materials and Methods

### Cell Lines and Cell Culture

SiHa (HTB-35), CaSki (CRL-1550) and HeLa (CCL-2) cell lines were obtained directly from the American Type Culture collection (ATTC, Manassas, VA, United States). C33A cells were kindly donated by Dr. Priscilla Brebi, La Frontera University, Temuco, Chile. The cells were incubated in RPMI1640 basal medium (Gibco, Carlsbad, CA, United States) supplemented with 10% fetal bovine serum (FBS) (Hyclone, Fremont, CA, United States) with antibiotics (penicillin and streptomycin) and maintained at 37°C with 5% CO_2_ atmosphere. For subculture, the cells were incubated with trypsin for 3–5 min and maintained with new medium containing FBS (Hyclone, Fremont, CA, United States). The cells were periodically tested for mycoplasma contamination.

### Real-Time Quantitative PCR

Following CSC treatment, the cells were homogenized with TRIzol reagent (Invitrogen; Thermo Fisher Scientific, Inc.). A total of 0.2 mL chloroform was then used to separate the upper phase that contained total RNA. The RNA samples were precipitated using isopropyl alcohol for 10 min and washed with 75% ethanol. All the RNA samples were resolved in nuclease free water (Promega Corporation, Madison, WI, United States). The RNA was treated with RQ1 RNase-free DNase (Promega, Madison, WI, United States) at 37°C for 60 min and then incubated with RQ1 DNase Stop Solution for 10 min. The cDNA was prepared using a 20 μL reaction volume containing DNAse-treated RNA (2 μg), 1 U RNAse inhibitor (Promega, Madison, WI, United States), 0.04 μg/μL random primers (Promega, Madison, WI, United States), 2 mM dNTP (Promega, Madison, WI, United States) and 10 U Moloney Murine Leukemia Virus (MMLV) reverse transcriptase (Promega, Madison, WI, United States). The reaction mixture was incubated for 1 h at 37°C. The cDNA was subjected to Real-time PCR quantification of gene expression with specific primers described in Table 1 in RotorGene 6000 system (Corbett Research, Sydney, NSW, Australia). Each qPCR volume was 25 μL in total and the components were as follows: 12.5 μL 2X SYBR Green Mastermix (Promega Corporation, Madison, WI, United States), 7.5 μL nuclease-free water and 1 μL cDNA template. The thermocycling conditions for qPCR were as follows: 94°C for 30 s, 58°C for 20 s and 72°C for 20 s, for a total of 40 cycles. The fold change was calculated using the 2^−ΔΔCt^ method.

**Table 1:** Primers used in this study.

Name	Primer forward	Primer reverse
E7	ATTTGCAACCAGAGACAACTG	CAATATTGTAATGGGCTCTGT
E6	CTGCAAGCAACAGTTACTGCG	TCACACACTGCATATGGATTC
FOS	AAGGAGAATCCGAAGGGAAAGG	GGCAATCTCGGTCTGCAAAG
JUN	GAGCTGGAGCGCCTGATAAT	CCCTCCTGCTCATCTGTCAC
CYP1B1	AACAAGGACCTGACCAGCAG	CCCTGAAATCGCACTGGTGA
ß-actin	AGCGAGCATCCCCCAAAG	GGGCACGAAGGCTCATCA
TRE M2	CCTGCACTGCTTGCCAACC	CGGTATTTAAGGCGTTGGCGC
TRE P1	CTCACCTAATTGCATAGTTGGC	CCCATGTGCAGTTTTACAAATG

### Dual-Luciferase Reporter Assay

SiHa and CaSki cells were plated in triplicate in 24-well plate at 70–80% confluency and were transfected with 500 ng pmir-Glo LCR luciferase reporter plasmid with 1 μL Lipofectamine 2000 per well (Invitrogen). The culture medium was replaced by Opti-MEM Reduced Serum. After 6 h incubation, the transfection mixture was replaced by culture medium containing 0.1% DMSO, 10 μg/mL for 24 h, the cells were rinsed in 1X PBS and harvested with Passive Lysis Buffer in a new tube on ice. Luciferase activity was measured using the Dual-Luciferase^®^ Reporter Assay System (Promega), according to the manufacturer’s protocol.

### Western Blotting

To determine the expression of E7, p53, pRb, c-Jun^S73^, c-Fos^T232^, EGFR, ERK and Akt, equal amount of protein (40 μg) obtained from vehicle- and CSC-treated cells was loaded on a 10% polyacrylamide gel (SDS-PAGE) and electrophoresed, followed by transfer to a nitrocellulose membrane. The membrane was blocked in 5% bovine serum albumin and incubated with primary antibody (1:500–1000 dilution) overnight. Next day, the membrane was washed and incubated with secondary antibody (1:2000 dilution). Membranes were washed three times in TBS-T and incubated with secondary anti IgG-labeled peroxidase (BD Pharmingen, San Diego, CA, United States). After washing three times in TBS-T, immune complexes were detected using the ECL system (Amersham Pharmacia Biotech) according to the manufacturer’s instructions. β-actin was used as an internal loading control to normalize the expression of all proteins.

### Viability Assays

The cervical cancer cells (SiHa, and CaSki) were cultured in 96 well plates at a density of 5 × 10^3^ cells/well. After 48 h, the cells were treated with CSC, DMSO, AG1478, U0126 at different concentrations and incubated for another 24–48 h. Viability was measured using the CellTitter 96^®^ AQueous non-radioactive Cell Proliferation Assay kit (Promega). Finally, it was added to each well 20 μL of the MTS reagent and the cells were incubated for 3 h. The absorbance of the product formazan salt was measured spectrophotometrically at 490 nm. The absorbance is proportional to the number of lives cells in each well.

### Immunofluorescence and Confocal Microscopy

1 × 10^5^ cells / well were seeded in Chambers Slides. After 24 h, when cells reached ∼60% confluency, were washed and the medium was replaced with medium containing CSC or their vehicle. Next, the cells were washed twice with PBS (pH 7.4), dried and incubated for 5 min with cold acetone. The fixed cells were then stored at −20°C until use. Cells were incubated with 1% bovine seroalbumin (BSA) for 30 min at room temperature, followed by incubation with a primary monoclonal anti-specific protein antibody diluted in PBS according to the manufacturer’s instructions. The fixed cells were washed three times for 5 min at room temperature and incubated with a secondary FITC-labeled anti IgG antibody. After three washes with PBS, the cells were visualized in a C2 Plus confocal microscope.

### Phospho MAPK Array

CaSki cells were grown to 90% confluence in 10 cm plates, subjected to serum starvation for 24 h and then treated with CSC (10 μg/mL) and equivalent DMSO concentration for 2 h. Cells were collected by centrifugation and washed once with PBS. The washed cell pellets were suspended in extraction lysis buffer and incubated with 20 min at 4°C. The protein concentration was determined using the Pierce BCA protein assay reagent according to the manufacture’s instruction. Screening for different proteins in cell lysates were performed with a Proteome profiler array kit (R&D Systems). For the parallel determination of the relative levels of phosphorylation of Mitogen-Activated Protein kinases and other serine/threonine kinases. The array allows to detect the relative phosphorylation of these kinases: Akt1, HSP27, p38 beta, Akt2, JNK1, p38 delta, Akt3, JNK2, p38 gamma, Akt pan, JNK3, p53, CREB, JNK pan, p70, S6K, ERK1, MKK3, RSK1, ERK2, MKK6, RSK2, GSK-3 alpha/beta, MSK2, TOR, GSK-3 beta, p38 alpha. Horseradish peroxidase substrate (Millipore Corporation, United States) was used to detect protein signal and data were captured by exposure to Fujifilm Light films. The analysis of Films was performed using the NIH ImageJ software.

### Chromatin Immunoprecipitation

CaSki cells were cultured in 10 cm dishes until 90% confluence, subjected to serum starvation for 24 h and treated 2 h with 10 μg/mL of CSC or DMSO. The cells were fixed by the addition of 1% formaldehyde for 5 min at room temperature. After the addition of 1 M glycine, cells were lysed with scraper and lysates were subjected to centrifugation at 6,000 rpm for 20 min at room temperature. After centrifugation, supernatants were removed and pellets were washed twice in A buffer (50 mM TRIS, pH 7,4, 200 mM NaCl, 1 mM EDTA, 0,1% Triton X-100, 0,1% NP-40, 0,01% SDS) and subjected to three pulses of sonication. The volume of sonicated chromatin was adjusted to 4 mL with A buffer – 0.1% SDS and then cleared by centrifugation at 13,000 rpm for 10 min. Chromatin was stored in 800-μL aliquots at –80°C. Immunoprecipitation of c-Jun^S73^ and c-Fos^T232^ bound to chromatin was carried out using preblocked immunoglobulin G (IgG)-Sepharose beads. Equal amounts of chromatin were incubated in the presence of 50 μL IgG-Sepharose beads in A buffer – 0.1% SDS. Before immunoprecipitation with IgG beads, 1% of each sample was saved as input fraction. The mixture was incubated for 2 h at 4°C on a rotary shaker. Beads were pelleted by centrifugation at 12.000 RPM for 1 min. 500 μL of immunoprecipitated were washed four times in 400 μL of A buffer and in 1 mL of TE buffer (10 mM Tris-HCl, pH 8.0, 1 mM EDTA), respectively. After each wash, the liquid suspensions were transferred to fresh tubes. Immunocomplexes were then eluted from the beads by two sequential incubations at 65°C for 10 min in 250 μL of elution buffer (50 mM Tris-HCl, pH 8.0, 10 mM EDTA, 1% SDS), followed by centrifugation. Eluted DNA and DNA of the input control were incubated first at 65°C for 18 h for de-cross-linking and subsequently in the presence of 50 μg proteinase K for 2 h at 37°C. Free DNA was purified from the solution by phenol-chloroform-isoamylalcohol (25:24:1) extraction. After centrifugation and precipitation by the addition of glycogen and ethanol, precipitated DNA was suspended in 100 μL of TE buffer for PCR analysis. PCR amplifications were performed by q-PCR in RotorGene 6000 system with an initial hold at 94°C for 5 min, follow of 25 cycles of 1 min at 94°C, 1 min at 60°C, and 2 min at 72°C and then 1 cycle of 4 min at 72°C. Primers that span the TRE sites proximal and the media distal region (named TRE P1 and TRE M2, respectively) inside the HPV16-LCR were used for PCR analysis.

### Anchorage Independent Growth

To analyze cell colony growth in soft agar, DMSO- and CSC-treated SiHa cells were trypsinized, thoroughly re-suspended, and re-plated at a density of 2500 cells in 4 mL of 0.3% Bacto agar (Becton Dickinson, Allschwil, Switzerland) over 0.6% agar base in 35- mm culture dishes, and covered with 2 mL of RPMI medium. The stock of Bacto agar was diluted with RPMI medium supplemented with 10% FBS, and penicillin/streptomycin. Colonies were visually and counted after 30 days of culture.

### Statistical Analysis

Data are expressed as the means ± standard deviations of three independent determinations. The significance of differences between the two samples was analyzed using Student’s *t*-test, and a p-value of < 0.05 was considered statistically significant.

## Results

### Tobacco Smoke Promotes HR-HPV E6/E7 Overexpression in Cervical Cancer Derived Cell Lines

Cigarette smoke condensate (CSC) is a complex mixture prepared in a “smoker chamber” using 3R4F standard cigarettes. This mixture contains particulate material with more that 4,000 compounds and approximately 60 carcinogens including nitrosamines and polycyclic hydrocarbons (Ewald et al., 2012). Therefore, it is expected that high concentrations of these compounds could be injurious for cells. In order to determine non-toxic CSC levels in cervical cancer derived cells, we exposed SiHa (2 copies HPV16/genome) and CaSki (500 copies HPV16/genome) cells to different concentrations of CSC. As observed in Supplementary Figures S1A,B, a dose of 10 μg/mL was the maximum non-toxic concentration because a non-significant viability change was observed. Consequently, this CSC concentration was selected for further experiments.

**FIGURE 1:**
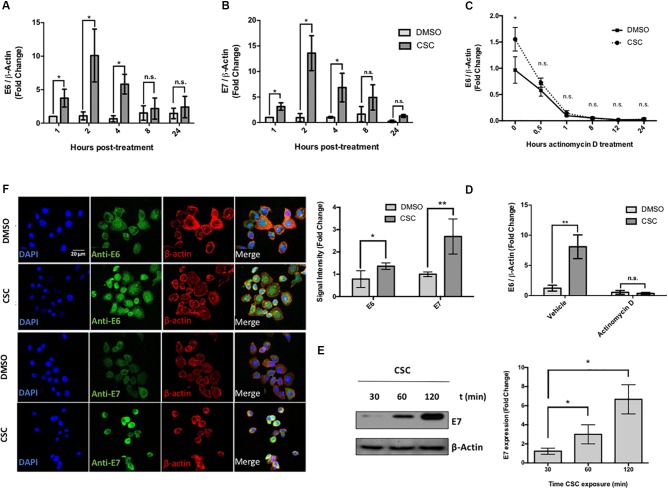
Cigarette smoke components promote an increase of E6/E7 levels in CaSki Cells. **(A,B)** CaSki cells were treated with 10 μg/mL CSC or DMSO at 1, 2, 4, 8, and 24 h. The obtained RNA was converted to cDNA and subjected to RT-qPCR with primers flanking HPV16 E6 **(A)** or E7 **(B)** oncogenes. The E6 and E7 transcript levels were normalized against ß-actin gene expression. **(C)** RT-qPCR with primers for E6 for RNA from CaSki cells treated with 10 μg/mL actinomycin D for different periods of time after exposure to CSC or DMSO. **(D)** RT-qPCR with primers for E6 transcripts from CaSki cells treated with 10 μg/mL actinomycin D or vehicle, after exposure to 10 μg/mL CSC and an equivalent DMSO concentration. **(E)** Immunoblot for E7 protein from CaSki cells exposed to 10 μg/mL CSC for different periods of time. **(F)** Confocal microscopy for E6 and E7 proteins in CaSki cells exposed for 24 h with 10 μg/mL CSC or DMSO using a secondary antibody conjugated to FITC fluorophore. The quantification of fluorescence signal intensity is shown to the side. DAPI: fluorescent marker of DNA, Rhodamine: fluorescent marker of β-actin cytoskeleton. Data shown are mean from three independent experiments. ^∗^*p* < 0.05, ^∗∗^*p* < 0.01, n.s: non-significant.

Previous reports, conducted *in vitro* and *in vivo*, demonstrated that CSC promotes CYP1B1 gene expression (Port et al., 2004; Nagaraj et al., 2006; Vidal et al., 2006). In order to confirm the biological activity of CSC used in this study, we evaluated CYP1B1 mRNA expression by RT-qPCR at different periods of time. As shown in Supplementary Figure S2A, CSC promotes a significant increase of CYP1B1 levels in CaSki cells at each analyzed time when compared to control. Further, CaSki and SiHa cells were exposed to CSC for different lengths of time (0 to 24 h) and E6 and E7 expression was analyzed at the RNA and protein levels. The CSC exposure significantly increased E6 (Figure 1A) and E7 (Figure 1B) levels with a maximum at 2 h post CSC incubation in CaSki cells. These results were also confirmed in SiHa cells, which reached a maximum level of E6/E7 at 4 h post CSC exposure (Supplementary Figures S2B,C). To discard the possibility of a differential E6/E7 transcripts decay rate after CSC or DMSO exposure, CaSki cells were exposed to 10 μg/mL actinomycin D, a known transcription inhibitor. As shown in Figures 1C,D, no significant difference in E6 transcripts decay was observed when CaSki cells were exposed to CSC and DMSO, suggesting that CSC leads to an increased E6 trancription. Similar results were obtained analyzing E7 transcripts (data not shown). We verified that in CaSki cells, E6 and E7 protein levels were significantly increased after CSC exposure, using western blot and immunofluorescence (Figures 1E,F). To determine the functional activity of E6 and E7 oncoproteins after CSC exposure, we evaluated p53 and pRB downregulation. As observed in Supplementary Figure S3A (left), the CSC treatment was associated with a decrease of p53 and pRB levels in CasKi cells. However, using the HPV negative cervical cancer cell line C33A, a decrease of p53 levels after CSC treatment was not observed (Supplementary Figure S3A right). In addition, *in vitro* tumor properties of SiHa cells exposed for 4 weeks to CSC were evaluated using soft agar. As shown in Supplementary Figure S3B, no significant changes were observed. Together, these results strongly suggest that CSC induces E6 and E7 overexpression in HPV16 positive cervical cancer cells which in turn, is associated with a decrease of p53 and pRB levels.

### Tobacco Smoke Induces LCR-Dependent p97 Promoter Activation in Cervical Cancer Derived Cell Lines

**FIGURE 2:**
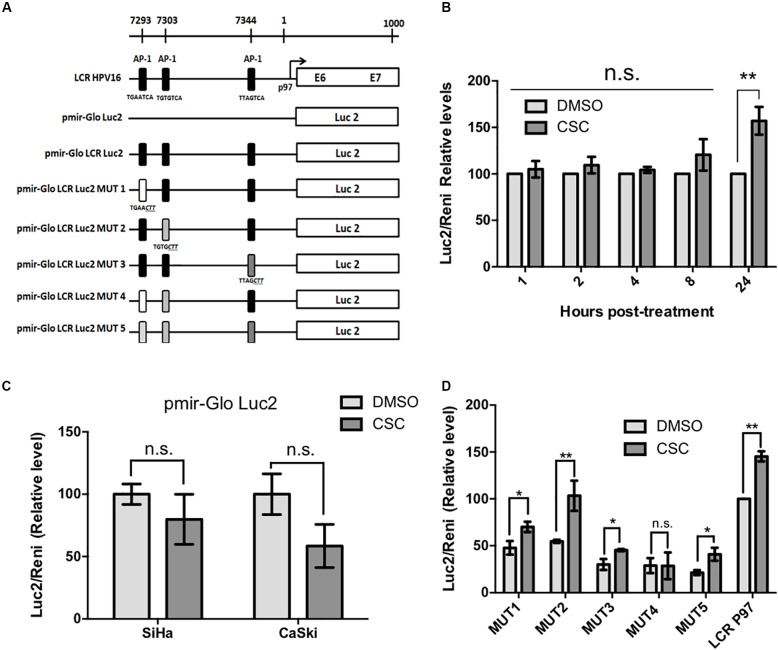
Cigarette smoke components induce HPV16 p97 promoter activity. **(A)** A schematic diagram depicts the different constructs used in this study. **(B)** Luciferase activity in CaSki cells previously transfected with pMIR-GLO LCR-Luc2 and treated with CSC or DMSO for different periods of time. **(C)** Luciferase activity in CaSki and SiHa cells transfected with pMIR-GLO-Luc2 exposed to 10 μg/mL CSC and an equivalent DMSO concentration for 24 h. **(D)** Luciferase activity of LCR mutants in CaSki subjected to CSC and DMSO treatment for 24 h. Data shown are mean from three independent experiments. ^∗^*p* < 0.05, ^∗∗^*p* < 0.01, n.s: non-significant.

The next step was to evaluate the p97 promoter activation by CSC in cervical cancer derived cell lines. For this, we transfected CaSki and SiHa cells with pMirGLO-LCR-p97-Luc2 construct, in which the HPV16 p97 promoter and LCR were located upstream of a luciferase reporter gene. A scheme showing the reporter constructs used in this study is shown in Figure 2A. We observed the maximum p97 promoter activation at 24 h post CSC-treatment (Figure 2B). In addition, removal of HPV16 LCR from the pMir-Glo-p97 promoter decreased luciferase activity upon CSC treatment (Figure 2C), suggesting that LCR activity is critical for CSC-mediated p97 promoter activation. Previous reports show that binding sites for the AP-1 transcription factors within the TREs of the LCR, are important for the activity of the HR-HPV early promoter (Chong et al., 1991). Therefore, in order to determine the relative importance of the three promoter-proximal AP-1/TRE sites for CSC-mediated p97 promoter activation, five TRE mutant versions of pMirGLO-LCR-p97-luc were obtained by site-directed mutagenesis (Figure 2A). As shown in Figure 2D, decreased p97 promoter activation was observed in TRE mutants 1, 3, 4, and 5. Moreover, a significant decrease in the activity was determined only with the mutants 3, 4, and 5. These results indicate that TRE sites located at positions 7344 (TTAGCTT) and 7293 (TGAACTT) are most important for CSC-mediated p97 promoter activation, suggesting a role for AP-1 binding.

### EGFR Activation and PI3K/Akt Signaling Pathway Is Involved in CSC-Mediated E6/E7 Overexpression

**FIGURE 3:**
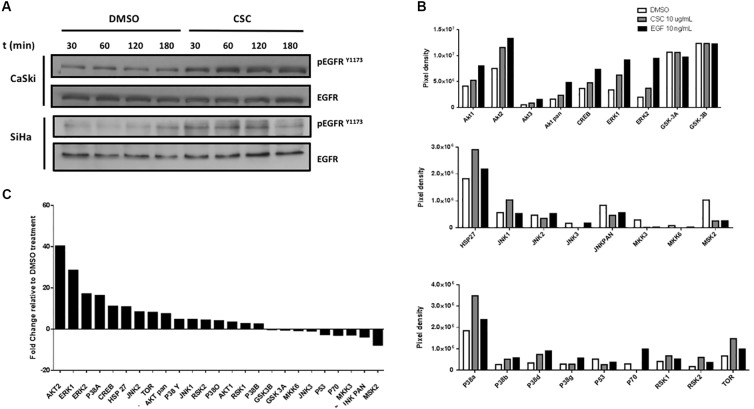
Cigarette smoke components induce the activity of MAPK and PI3K pathways. **(A)** Immunoblot with antibodies directed against phosphorylated EGFR at tyrosine 1173 (EGFR^Y 1173^) in CaSki and SiHa cells treated with DMSO or CSC for different periods of time. **(B)** Densitometric analysis of microarrays for protein members of the MAPK and PI3K pathways in CaSki cells treated with DMSO, CSC or EGF for 2h. The results are expressed as pixel density from spots of each protein type. **(C)** Fold change analysis for pixels from CSC relative to DMSO.

**FIGURE 4:**
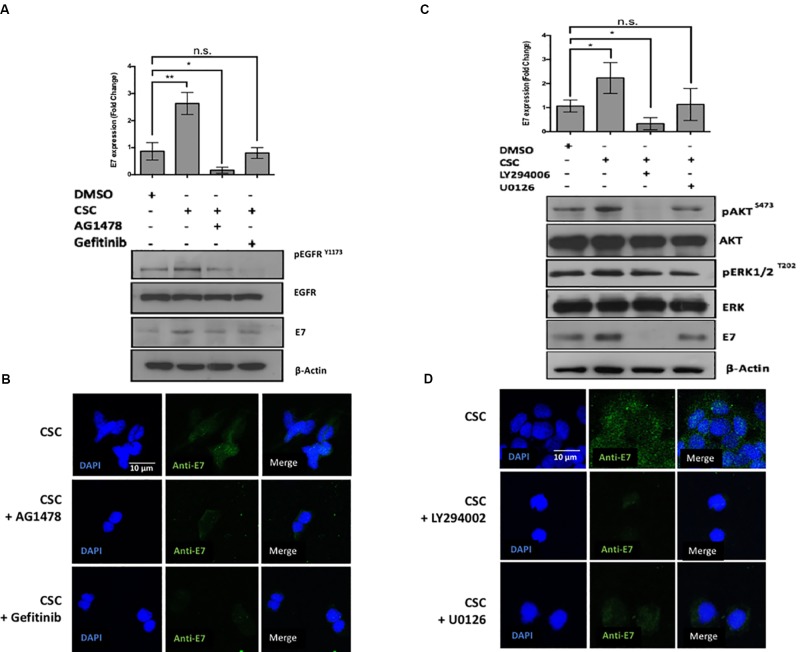
The loss of Akt signaling is associated with a decrease in E7 protein levels. **(A)** CaSki cells previously exposed to inhibitors against EGFR were incubated in RPMI medium containing DMSO or CSC for 2 h. The protein extracts were subjected to immunoblot directed against EGFR^Y 1173^ and E7. Above: quantification of E7 inmunoblot relative to β-actin levels. **(B)** Indirect immunofluorescence was made in CaSki cells with antibodies directed against E7. **(C)** CaSki cells previously exposed to chemical inhibitors against ERK, Akt, were incubated in RPMI medium containing DMSO or CSC. The protein extracts were immunoblotted with antibodies directed against pERK^T202^, pAkt^S473^ and E7. Above: quantification of E7 immunoblot relative to β-actin levels. **(D)** CaSki cells were treated with DMSO or CSC and subjected to indirect immunofluorescence with antibody directed against E7. Data are mean from three independent experiments.

Previous reports demonstrated that CSC promotes the EGFR activation and phosphorylation in bronchial, duct and pancreatic duct epithelia models (Lemjabbar et al., 2003; Askari et al., 2005; Martínez-García et al., 2008). Thus, we evaluated whether EGFR was activated by CSC in the cervical cancer cell-derived CaSki and SiHa cell lines. As observed in Figure 3A, an increase in EGFR phosphorylation at Y1173 was observed at 30 min after 10 μg/mL CSC exposure and it was sustained through 3 h. To assess the downstream targets activated by EGFR signaling in response to CSC, a phosphoproteomics approach was used (Supplementary Figure S4). When CaSki cells were exposed to CSC, increases in phosphorylated forms of Akt1, Akt2, CREB, Erk1, Erk2, Jnk1, p38a, p38d and mTOR phosphorylation were observed (Figure 3B). Conversely, phosphorylation of JNK/pan, MKK3, Msk2, p53, and p70 was decreased after CSC exposure. As observed in Figure 3C, after normalizing against the DMSO-treated cells, increases in phosphorylated Akt2 and Erk1 were the most pronounced after CSC treatment, suggesting the PI3K and MAPK pathways were activated downstream of EGFR. Since CSC treatment stimulated E6/E7 overexpression in cervical cancer cells and concomitantly induced PI3K and MAPK activation, we used specific inhibitors to verify the importance of these signaling pathways for inducing an increase of oncogene levels in CaSki cells. As shown in Figures 4A,B, CSC promoted an increase of E7 protein levels which in turn, was abrogated in the presence of the EGFR inhibitors, AG1478 and gefitinib. Additionally, the PI3K inhibitor LY294002 blocked CSC-mediated activation of phospho-Akt (S473), and completely abolished expression of E7, suggesting that PI3K/Akt signaling is critical for CSC-mediated activation of the HPV16 early promoter. Finally, the MEK inhibitor U0126 decreased the activation of phospho-ERK1/2 by CSC, and also prevented the increase in E7 expression (Figures 4C,D). These experiments confirmed the importance of EGFR activation leading to PI3K signaling for CSC-mediated E7 overexpression, and indicated that MEK activation also plays a role therein.

### JUN Is Overexpressed, Phosphorylated and Interacts With TRE/AP-1 Sites in the HPV16 LCR in CSC-Exposed Cells

**FIGURE 5:**
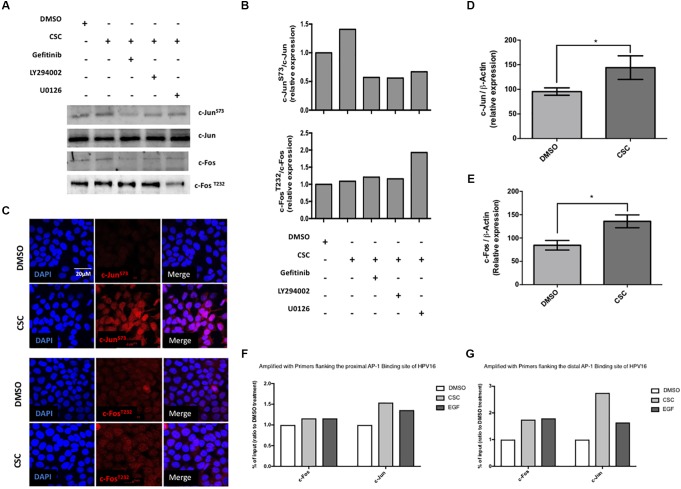
Cigarette smoke components induce phosphorylation of c-Jun but not c-Fos in CaSki cells. **(A)** CaSki cells previously exposed to inhibitors against EGFR, PI3K and ERK were incubated in RPMI medium containing CSC. The protein extracts were subjected to immunoblot with antibodies directed against c-Jun^S73^ or c-Fos ^T232^ **(B)** quantification of c-Jun^S73^ and c-Fos ^T232^ immunoblots relative to total protein levels **(C)** Confocal microscopy for c-Jun^S73^ and c-Fos^T232^ in CaSki cells treated with DMSO or CSC for 2 h, using a secondary antibody conjugated to the Texas Red fluorophore. DAPI: fluorescent DNA marker. **(D,E)** RT-qPCR with primers for c-Jun or c-Fos from total lysates of CaSki cells subjected to treatment with CSC or an equivalent DMSO concentration for 24 h. **(F,G)** ChIP assay detected the binding of c-Jun and c-Fos to the proximal and media distal region in the HPV16-LCR post CSC treatment. ^∗^*p* < 0.05.

Experiments shown in Figure 2 demonstrated the importance of AP-1 binding sites (TRE) in the HPV16 LCR for CSC-mediated p97 promoter activation. Therefore, we evaluated the levels of JUN and FOS transcripts and the phosphorylation levels of c-Jun and c-Fos proteins in CaSki cells exposed to CSC. An increased c-Jun phosphorylation (S73) was observed upon CSC treatment additionally, the use of specific inhibitors against EGFR and PI3K was related to c-Jun^S73^ decrease (Figures 5A,B). By indirect immunofluorescence we found c-Jun^S73^ in the nucleus of CaSki cells (Figure 5C) and SiHa cells (Supplementary Figure S5). However, we did not detect significant changes in c-Fos phosphorylation (T232) after CSC exposure (Figures 5A,B). On the other hand, a significant increase in JUN and FOS transcripts was detected after 24 h of CSC exposure of CaSki cells (Figures 5D,E). In order to further address this issue, we conducted a chromatin immunoprecipitation (ChIP) using antibodies for c-Fos and c-Jun enrichment on the two key CSC-activated TRE/AP-1 sites in the LCR. In CaSki cells exposed to CSC, we found enrichment of both c-Fos and c-Jun on the distal TRE/AP-1 site (Figure 5F). Much less enrichment of c-Jun, and little if any of c-Fos enrichment was detected on the proximal TRE AP-1 site after CSC exposure (Figure 5G). Taken together, our data strongly suggest that activated c-Jun is a mediator of CSC-induced E6/E7 overexpression in CaSki cells.

## Discussion

It has been clearly established that HR-HPV infection is not a sufficient condition for cervical cancer development (Schiffman et al., 2007). Tobacco smoking is a known cofactor involved in the development of this disease and others (Louie et al., 2011). Here, we addressed the mechanisms and signaling pathways involved in the interplay between HR-HPV and tobacco smoking leading to E6 and E7 overexpression. It is necessary to consider that a potential interaction between these two carcinogenic agents in the population is very complex due to additional host, environmental and viral-associated factors (Szarewski et al., 2001; Wiley et al., 2006). Here, we focused only on E6 and E7 oncoproteins because are the only viral factors expressed in all of the HR-HPV associated cancers and their sustained expression is a necessary condition for HR-HPV-mediated cancer initiation and progression (zur Hausen, 2000).

In this study, we found that tobacco smoke promotes an increase of E6 and E7 oncoproteins levels in cervical cancer-derived cell lines. In addition, we found that CSC-mediated E6/E7 overexpression is carried out by p97 promoter activation in cervical cancer-derived cells in a dose-dependent manner. Previously, we found that p97 promoter activation occurs in other epithelial tumor cells such as lung or oral (Pena et al., 2015). Here, we found that this early promoter activation depends on the presence of an intact LCR. A number of *in vitro* studies have been conducted to determine the importance of AP-1 binding sites into the LCR for regulation of the early promoter of HPV16 (Chan et al., 1991; Chong et al., 1991; Kyo et al., 1997). Furthermore, it has been previously demonstrated that CSC exposure promotes an increase of AP-1 activity in several cell models (Swenson et al., 2011; Wang et al., 2017). In this study, we evaluated the importance of TRE sites using specific mutants and we discovered that specific mutants in the proximal TRE sites significantly affect the CSC-induced p97 promoter activity. Previous studies in fibroblasts have shown that the proximal AP-1 site is required for transactivation of the LCR of HPV16 during the viral life cycle (Kikuchi et al., 1996). However, other studies have shown that distal AP-1 site is critical for enhancer function whereas the proximal AP-1 site seems to be important only in certain cell types (Bauknecht et al., 1992). Thus, it is plausible that AP-1 might be part of the molecular mechanisms mediating CSC-induced E6 and E7 overexpression. Nonetheless, since cigarette smoke contains more than 4,000 different chemical compounds, including abundant reactive oxygen/nitrogen species and aldehydes, and many other carcinogens, we cannot rule out the possibility that other signaling pathways can be involved in an orchestrated manner for inducing E6/E7 up-regulation. Indeed, it has been shown that cigarette smoke exposure mediates global signaling changes in lung cells, influencing multiple events, including cell polarity, cytoskeletal remodeling, cell migration, protein synthesis, autophagy, and apoptosis (Mathis et al., 2013; Solanki et al., 2017).

In this study, we showed that by inhibiting EGFR with gefitinib in CSC-treated CaSki cells, there was an evident decrease in E7 levels. However, treatment of the same CSC-exposed cells with U0126, which inhibits MEK protein phosphorylation, failed to induce significant changes. These findings suggest that MEK phosphorylation, although is activated by CSC, is not essential to induce overexpression of E6/ E7 oncogenes. Previously, it has been shown that MAPK pathway is involved in HPV31 replication by inducing the activity of the HPV31 late promoter (Bowser et al., 2011). On the other hand, the Akt inhibition with LY294002, completely abolished E7 levels, suggesting a critical role of this signaling pathway for CSC-induced HPV16 oncogenes overexpression. The PI3K/Akt/mTOR signaling controls most hallmarks of cancer: cell cycle, survival, metabolism, motility, and genomic instability (Hanahan and Weinberg, 2011). It has been demonstrated that PI3K is amplified and activated in HR-HPV-induced cervical cancers (Lee et al., 2006), and plays a very important role in HPV-induced carcinogenesis by acting through a plethora of cellular and molecular events (Zhang et al., 2015). Previous studies have shown that the E7 protein of HPV16 augments the activation of Akt through the retinoblastoma protein, promoting an increase in downstream signaling (Menges et al., 2006). On the other hand, enhanced Akt signaling promotes keratinocyte differentiation, and pharmacologic inhibition of Akt signaling reduces keratinocyte differentiation. Therefore, it seems that activation of PI3K signaling favors the HPV16 life cycle. Also, it has been described that PI3K signaling pathway is a mediator of cigarette effects. For instance, nicotine stimulation promotes cell growth and survival by activating nicotinic acetylcholine receptors (nAChRs) via PI3K-Akt (Takeuchi et al., 2009). In addition, some nitrosamines such as 4-(methylnitrosamino)-1-(3-pyridyl)-1-butanone or NNK are associated to the formation of DNA, hemoglobin or lipid adducts, through also trough PI3K. Therefore, this signaling pathway, activated by cigarette components can change the cell fitness promoting the oncogenes overexpression (Zhang et al., 2006) histone phosphorylation, and cell transformation (Ibuki et al., 2014).

The AP-1 transcription factor participates in the control of cellular responses to stimuli that regulate proliferation, differentiation, immune response, cell death and the response to genotoxic agents or stress (Mechta-Grigoriou et al., 2001). The molecular composition of the AP-1 complex is heterogeneous. Both the Fos and Jun families of nuclear phosphoproteins may participate in the formation of homo or heterodimeric complexes (Kaminska et al., 2000). Our results showed that JUN expression and protein phosphorylation increase after CSC-treatment. In agreement with this finding, previous studies have also shown that different members of AP-1 complex such as c-Fos or c-Jun are activated and are able to promote gene expression during cigarette smoke exposure (Geng et al., 2017; Yu et al., 2017). However, in this study we did not observe changes in c-Fos phosphorylation status. Using supershift analysis, many studies demonstrated that composition of the AP-1 complex is different under various physiological and pathophysiological conditions. In fact, closely related members of the same family might contribute to quite distinct biological phenomena (Pyrzynska et al., 2000). Thus, it is possible that in our model the compositional changes of AP-1, due the cell cycle or associated with the CSC treatment, promote an increase and activation of c-Jun complexes. On the other hand, c-Fos and c-Jun heterodimerize through their leucine zippers to form the AP-1 transcription factor. It has been reported that c-Fos stability decrease when the protein is dimerized with phosphorylated c-Jun (Tsurumi et al., 1995), suggesting the possibility that c-Fos activation can be impaired by c-Jun activity and subsequently leading to an increase of phospho c-Jun. Further experiments are necessary to explore this possibility.

**FIGURE 6:**
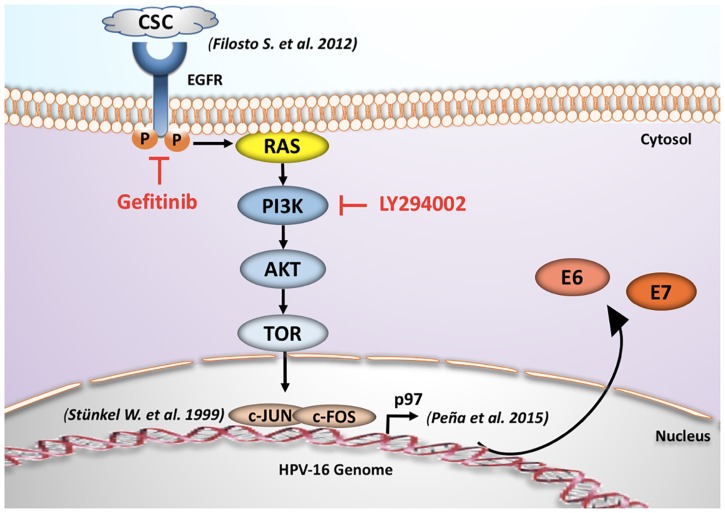
Proposed mechanism for tobacco smoking-mediated E6/E7 overexpression. The acute exposure to cigarette smoke condensate increases the transcription of early HPV16 E6 and E7 genes through EGFR/PI3K/Akt/AP-1 signaling pathway and subsequent p97 promoter activation in CaSki cells (Stünkel and Bernard, 1999; Filosto et al., 2012).

In summary, our results suggest an interplay between cigarette smoke exposure and HPV16 through EGFR-PI3K-AP-1 signaling that favors p97 activity and E6 and E7 overexpression in CaSki and SiHa cells. In addition, c-Jun activation and AP-1 binding sites are important for p97 promoter activation by tobacco smoke in cervical cancer-derived cells. A model about HPV and tobacco smoke interplay is proposed in Figure 6.

## Author Contributions

JM designed and performed all of the experiments and contributed with manuscript writing. DC-B and VA contributed with discussion of results and design of some experiments. OL, EM, GC, EB, JT, and MO contributed with discussion of results and manuscript writing. FA provided overall guidance, design of experiments, discussion and manuscript writing.

## Conflict of Interest Statement

The authors declare that the research was conducted in the absence of any commercial or financial relationships that could be construed as a potential conflict of interest.

## Supplementary Material

FIGURE S1 Cigarette smoke components induce a loss of cell viability in a concentration-dependent manner. (A,B) Viability of CaSki and SiHa cells exposed to cigarette smoke condensate (range: 0–100 μg/mL) for 72 h evaluated through MTS incorporation. Data shown are mean from three independent experiments. ^∗^*p* < 0.05.

FIGURE S2 CSC exposure increases CYP1B1, E6 and E7 expression levels in cervical cell lines. (A) CYP1B1 mRNA was evaluated through RT-qPCR after CSC treatment in CaSki cells. (B,C) E6 and E7 mRNA levels were evaluated in SiHa cells at different times of CSC exposure using RT-qPCR. ^∗^*p*< 0.05, n.s: non-significant.

FIGURE S3 Treatment with cigarette smoke components induce changes in the cell phenotype. (A) CaSki cells were treated with 10 μg/mL CSC or DMSO for different periods of time using cycloheximide as a translation inhibitor. The protein extracts were used for immunoblotting with antibodies directed against p53 and pRB. (B) Soft agar from SiHa cells exposed to DMSO or CSC. The images are representative of three independent experiments.

FIGURE S4 Human Phospho MAPK array. CaSki human cervical epithelial carcinoma cells were treated with DMSO, CSC or EGF for 2 h before lysis. The membranes were incubated with 200 μg of lysate according to the manufacturer’s instructions. Data shown are from a 2-min exposure to X-ray film. Each membrane contains 26 different capture antibodies printed in duplicate.

FIGURE S5 Cigarette smoke components induce c-Jun phosphorylation in SiHa cells. Confocal microscopy for c-Jun^S73^ and c-Fos^T232^ in SiHa cells treated 2 h with DMSO and CSC, using a secondary antibody conjugated to the Texas Red fluorophore. DAPI: fluorescent DNA marker, Rhodamine: fluorescent marker of cytoskeleton of actin. The images are representative of three independent experiments.
