# Supplementary figures and images for: Tobacco Exposure Enhances Human Papillomavirus 16 Oncogene Expression via EGFR/PI3K/Akt/c-Jun Signaling Pathway in Cervical Cancer Cells
Source: Front Microbiol. 2018 Dec 17;9:3022. doi: 10.3389/fmicb.2018.03022 (PMC6304352; doi:10.3389/fmicb.2018.03022)

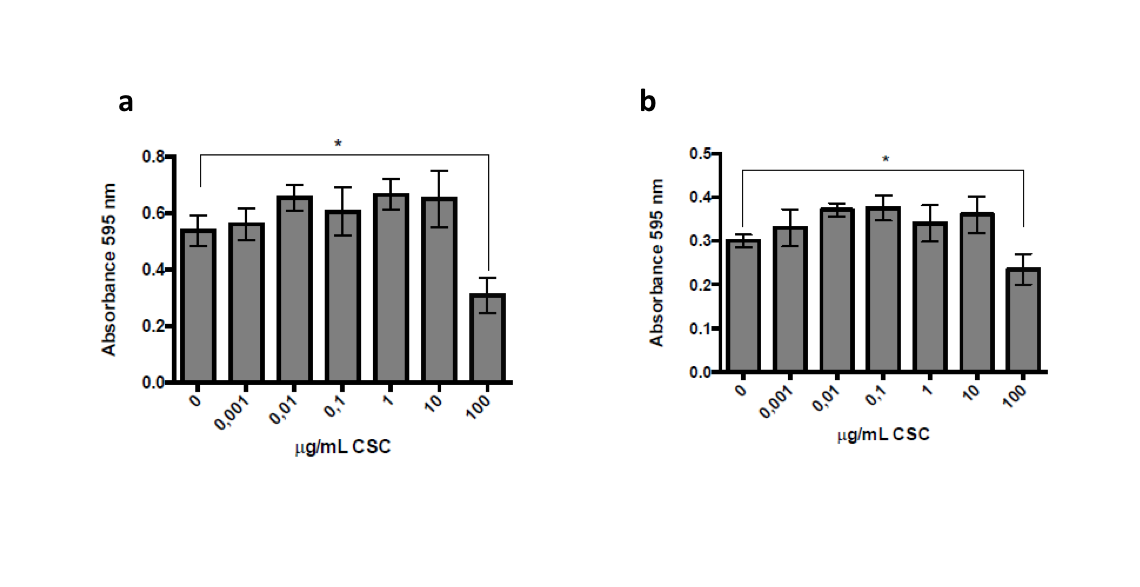

Supplement: FIGURE S1 — Cigarette smoke components induce a loss of cell viability in a concentration-dependent manner. (A,B) Viability of CaSki and SiHa cells exposed to cigarette smoke condensate (range: 0–100 μg/mL) for 72 h evaluated through MTS incorporation. Data shown are mean from three independent experiments. ∗p < 0.05. [file Image_1.TIF]

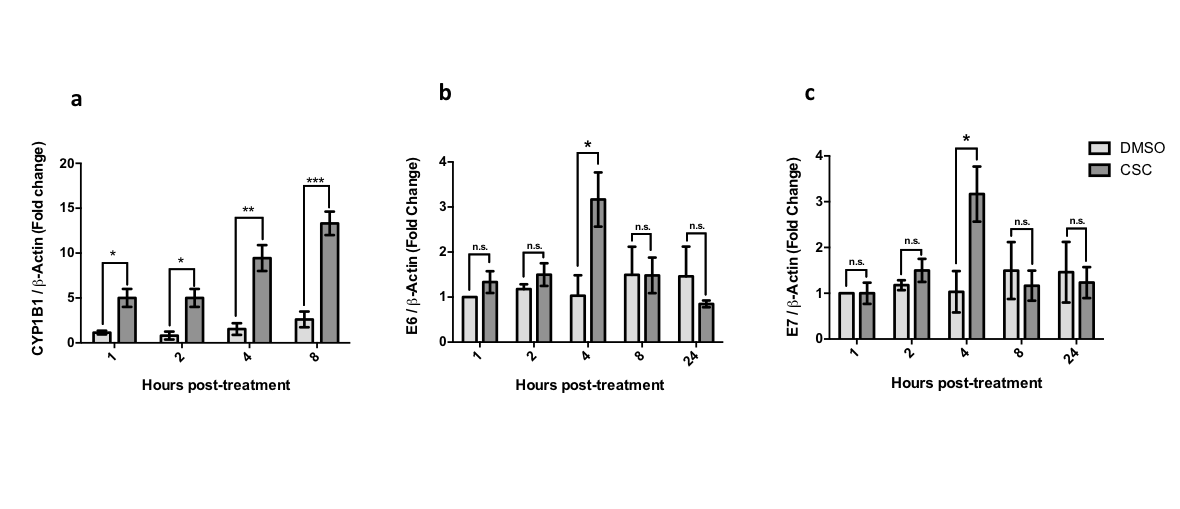

Supplement: FIGURE S2 — CSC exposure increases CYP1B1, E6 and E7 expression levels in cervical cell lines. (A) CYP1B1 mRNA was evaluated through RT-qPCR after CSC treatment in CaSki cells. (B,C) E6 and E7 mRNA levels were evaluated in SiHa cells at different times of CSC exposure using RT-qPCR. ∗p< 0.05, n.s: non-significant. [file Image_2.TIF]

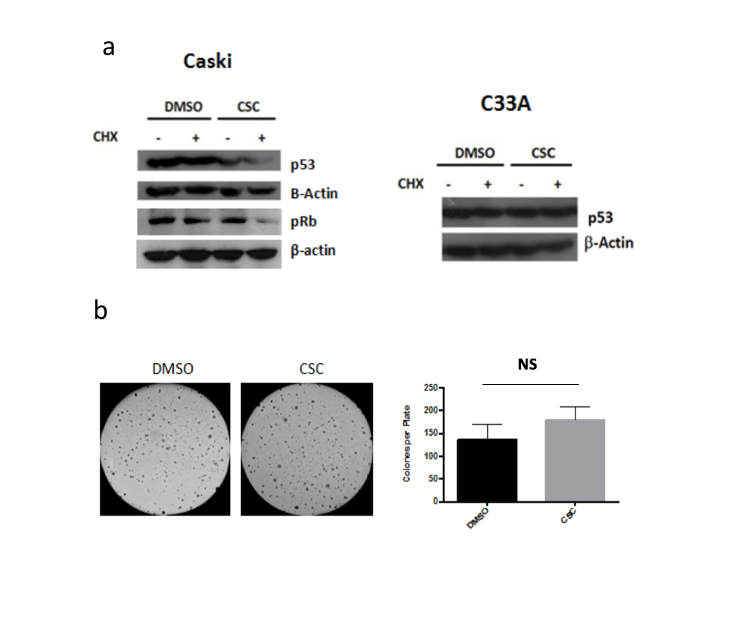

Supplement: FIGURE S3 — Treatment with cigarette smoke components induce changes in the cell phenotype. (A) CaSki cells were treated with 10 μg/mL CSC or DMSO for different periods of time using cycloheximide as a translation inhibitor. The protein extracts were used for immunoblotting with antibodies directed against p53 and pRB. (B) Soft agar from SiHa cells exposed to DMSO or CSC. The images are representative of three independent experiments. [file Image_3.TIF]

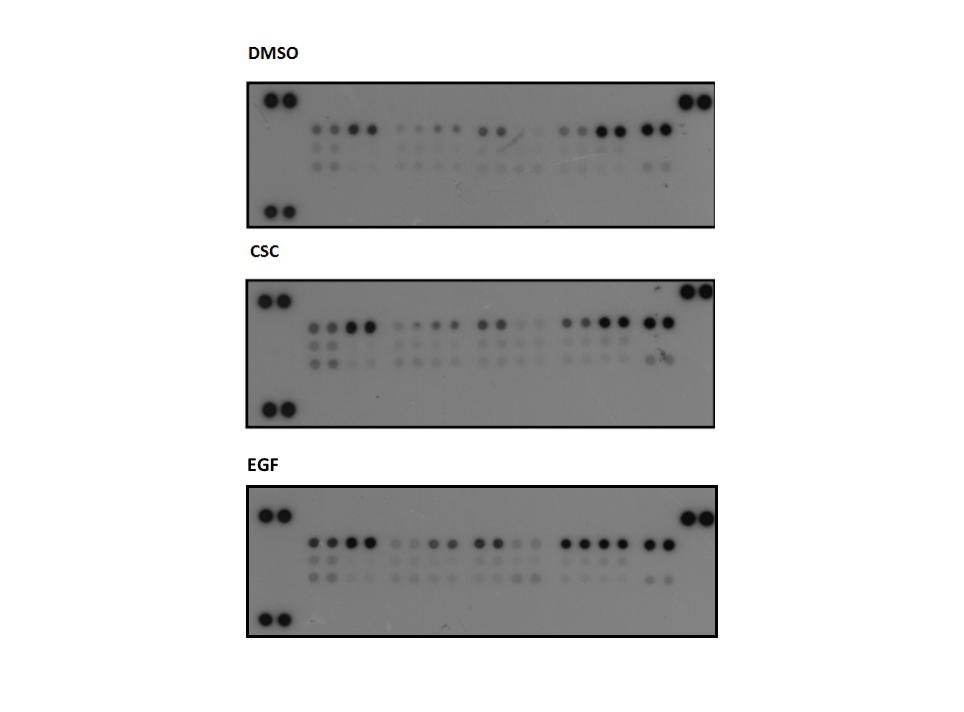

Supplement: FIGURE S4 — Human Phospho MAPK array. CaSki human cervical epithelial carcinoma cells were treated with DMSO, CSC or EGF for 2 h before lysis. The membranes were incubated with 200 μg of lysate according to the manufacturer’s instructions. Data shown are from a 2-min exposure to X-ray film. Each membrane contains 26 different capture antibodies printed in duplicate. [file Image_4.TIF]

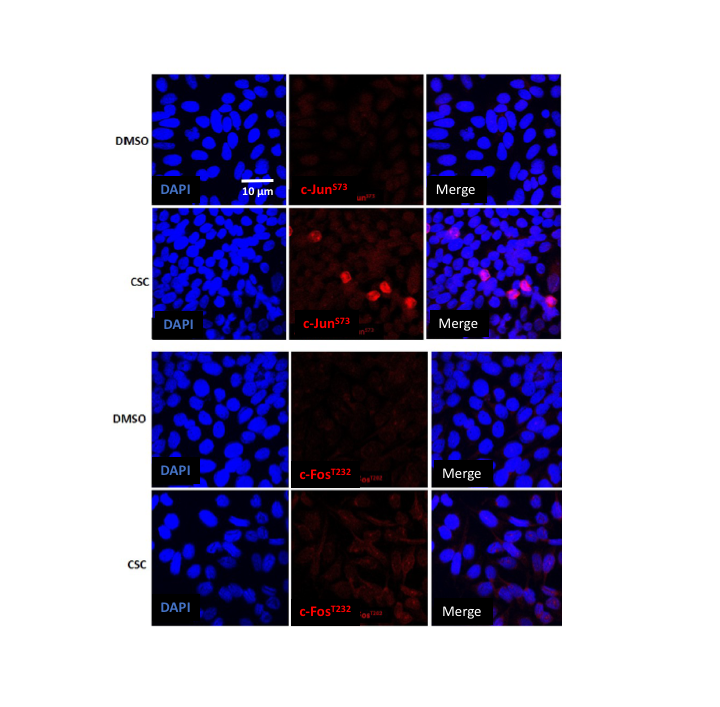

Supplement: FIGURE S5 — Cigarette smoke components induce c-Jun phosphorylation in SiHa cells. Confocal microscopy for c-JunS73 and c-FosT232 in SiHa cells treated 2 h with DMSO and CSC, using a secondary antibody conjugated to the Texas Red fluorophore. DAPI: fluorescent DNA marker, Rhodamine: fluorescent marker of cytoskeleton of actin. The images are representative of three independent experiments. [file Image_5.TIF]
